# Clinical and Electrophysiological Heterogeneity of Orthostatic Tremor: An Individual Patient Data Analysis

**DOI:** 10.5334/tohm.1278

**Published:** 2026-10-08

**Authors:** Leroy D’Souza, B. R. Niharika, Julián Benito-León, B. R. Samarth, Rishika Das, Muskan Kotwal, Kruthika Reddy, Pranav Anil Katti, C. S. Pranav Bhat

**Affiliations:** 1 Bangalore Medical College and Research Institute, Bangalore, India; 2 12 de Octubre University Hospital, Madrid, Spain; 3 Instituto de Investigación Sanitaria Hospital 12 de Octubre (imas12), Madrid, Spain; 4 Centro de Investigación Biomédica en Red Sobre Enfermedades Neurodegenerativas (CIBERNED), Madrid, Spain; 5 Complutense University of Madrid, Madrid, Spain

**Keywords:** Orthostatic tremor, Shaky leg syndrome, Primary orthostatic tremor, Orthostatic tremor plus, Pseudo-Orthostatic tremor, Electromyography

## Abstract

**Background:**

Orthostatic tremor (OT) is traditionally classified using tremor frequency and the presence of associated neurological signs. However, increasing reports of overlapping frequencies, fluctuating electrophysiological profiles, and diverse clinical associations indicate that current classification systems may not fully capture the clinical heterogeneity of OT.

**Methods:**

A systematic review of PubMed/MEDLINE from inception to February 2026 identified case reports, case series, and original studies reporting electromyography-confirmed OT with individual patient data. Demographic, clinical, electrophysiological, and data on associated conditions were extracted. Cases were classified according to the 2018 Movement Disorder Society criteria and separately grouped as isolated or non-isolated OT. Associations between tremor frequency and clinical phenotype were assessed using receiver operating characteristic analysis and multivariable logistic regression.

**Results:**

One hundred studies comprising 244 patients were included. Mean tremor frequency was 13.32 ± 4.15 Hz (range 3–20 Hz). Seventeen patients (6.97%) could not be classified using current criteria because of frequencies exceeding 18 Hz or fluctuations across the 13-Hz threshold. Non-isolated OT had significantly lower tremor frequencies than isolated OT (11.03 Hz vs 14.80 Hz, p < 0.001). Tremor frequency independently predicted non-isolated phenotype (OR 0.78) (AUC 0.72). Parkinsonian disorders were the most common associated condition, but substantial frequency overlap was observed across neurological and systemic disorders associated with OT.

**Discussion:**

Most cases were classifiable using the current OT criteria. However, a small subset demonstrated boundary-spanning or out-of-range frequencies. These findings suggest that tremor frequency remains an important electrophysiological feature of OT but should be interpreted alongside the clinical context.

**Highlights:**

Electromyography-confirmed orthostatic tremor exhibited marked clinical and electrophysiological heterogeneity across neurological and non-neurological conditions. Most cases were classifiable using the current OT criteria. However, a small subset demonstrated boundary-spanning or out-of-range frequencies. These findings suggest that tremor frequency remains an important electrophysiological feature of OT but should be interpreted alongside the clinical context.

## Introduction

Orthostatic tremor (OT), also known as “shaky legs syndrome,” was first described as a distinct entity by Heilman [1], although similar cases had been reported previously [2]. OT is characterized by unsteadiness and leg shaking while standing, with symptoms typically improving after sitting or walking [3]. Although the legs are predominantly involved, upper-limb, truncal, and facial muscle involvement has also been reported. Compared with more common tremor syndromes, such as essential tremor, OT remains rare [4,5].

The diagnosis of OT requires electrophysiological confirmation with electromyography (EMG) [6]. Historically, a standing tremor frequency of 13–18 Hz was considered essential for diagnosis [7]. Using this criterion, Gerschlager et al. [8] divided OT into two categories: primary OT and OT plus, the latter referring to OT associated with other neurological conditions. Several alternative classification schemes have subsequently been proposed, including distinctions based on tremor frequency (slow, intermediate, and classical OT) and association with neurological disorders, reflecting uncertainty regarding the optimal framework. [9,10,11].

The 2018 consensus statement of the International Parkinson and Movement Disorder Society (MDS) classified tremor syndromes along two axes: Axis 1, based on clinical features, and Axis 2, based on etiology. Within this framework, OT was classified as primary orthostatic tremor (13–18 Hz and isolated), primary orthostatic tremor plus (13–18 Hz with additional neurological signs), or pseudo-orthostatic tremor (<13 Hz, often associated with other neurological conditions) [6].

Despite these classification systems, several limitations remain. Primary OT is defined by a 13–18 Hz frequency range, yet cases with frequencies above 18 Hz have also been reported [12,13,14,15]. Frequency may also fluctuate over time or within the same recording, making classification dependent on the timing and duration of measurement [9]. Overlap between intermediate (10–13 Hz) and classical frequency ranges further blurs subtype distinctions [16]. Additionally, isolated cases in the intermediate range appear to share more features with primary (classical) OT (≥13 Hz) than with other slow tremor syndromes [17,18]. In addition, the distinction between primary OT, OT-plus, and pseudo-orthostatic tremor may not always be straightforward, with associated non-neurological conditions not represented. The current classification also may not adequately account for this possible longitudinal progression, where isolated presentations may progress to more complex or secondary forms over time [19].

Given these limitations, it remains uncertain whether current classification frameworks fully capture the clinical heterogeneity of OT. In particular, rigid frequency thresholds and a binary distinction between primary OT and OT-plus may not adequately reflect cases with fluctuating or boundary-spanning frequencies or non-neurological systemic associations. Hence, there is a need to evaluate whether frequency-based and etiological classifications adequately reflect the observed clinical heterogeneity in published cases of OT. The present review therefore evaluates the relationships between tremor frequency, associated conditions, and clinical parameters to better characterize the clinical and electrophysiological heterogeneity through a systematic review of published case reports. We hypothesize that orthostatic tremor represents a heterogeneous disorder and that its clinical expression is better captured through an integrated framework incorporating clinical phenotype and frequency variability.

## Methods

This systematic review was conducted in accordance with the Preferred Reporting Items for Systematic Reviews and Meta-Analyses (PRISMA) guidelines.

### Search Strategy

A comprehensive search of PubMed/MEDLINE was conducted from database inception through February, 2026. The search string used was: (“orthostatic tremor”) OR (“shaky leg syndrome”) OR (“shaky legs syndrome”) OR (“slow orthostatic tremor”) OR (“pseudo-orthostatic tremor”) OR (“orthostatic tremor plus”) OR (“secondary orthostatic tremor”).

### Selection Criteria

We included case reports, case series, and original studies that provided individual patient data for patients with EMG-confirmed OT, defined as rhythmic lower-limb muscle activity occurring during standing. Outcomes of interest included demographic data, OT frequency, and other associated conditions/disorders at presentation. Studies were excluded if they did not provide individual patient data or did not report or measure OT frequency. Orthostatic myoclonus cases were excluded.

### Data Extraction

Two reviewers independently screened the titles and abstracts of all identified articles after deduplication. Full-text articles of potentially relevant studies were retrieved and assessed for eligibility. Each case was classified according to the MDS OT classification [6]. In addition, cases were operationally classified as isolated or non-isolated based on associated conditions at presentation. This study grouping was used for analysis and was not intended to establish causality. When frequency was reported as a range for a single patient, the average of the range was taken for analysis.

### Statistical Analysis

Statistical analyses were performed using IBM SPSS Statistics, version 22.0 (IBM Corp., Armonk, NY, USA). Normality was assessed by visual inspection of histograms and Shapiro-Wilk tests. Continuous variables are presented as mean (SD), and categorical variables are summarized as frequencies and percentages. Receiver operating characteristic (ROC) analysis was used to evaluate the ability of tremor frequency to distinguish isolated from non-isolated OT presentations. The Youden index was calculated to determine the optimal frequency threshold. Spearman’s correlations, Chi-square and Mann-Whitney U tests were used to assess associations between variables. A p value < 0.05 was considered statistically significant.

## Results

### Cohort characteristics and clinical Profile

A total of 100 articles fulfilled the inclusion criteria and were included in the analysis (Figure 1), comprising 244 patients (Supplementary table 1 and References [9,10,12,13,14,15,17,20,21,22,23,24,25,26,27,28,29,30,31,32,33,34,35,36,37,38,39,40,41,42,43,44,45,46,47,48,49,50,51,52,53,54,55,56,57,58,59,60,61,62,63,64,65,66,67,68,69,70,71,72,73,74,75,76,77,78,79,80,81,82,83,84,85,86,87,88,89,90,91,92,93,94,95,96,97,98,99,100,101,102,103,104,105,106,107,108,109,110,111,112]). The cohort had a mean age of 62.61 ± 13.43 years. Age ranged from 7 to 86 years. A female predominance was observed with 96 male patients and 148 female patients. A positive family history of OT was present in 17 patients. Mean disease duration among cases which reported the same (n = 222) was 7.83 ± 6.72 years.

**Figure 1:**
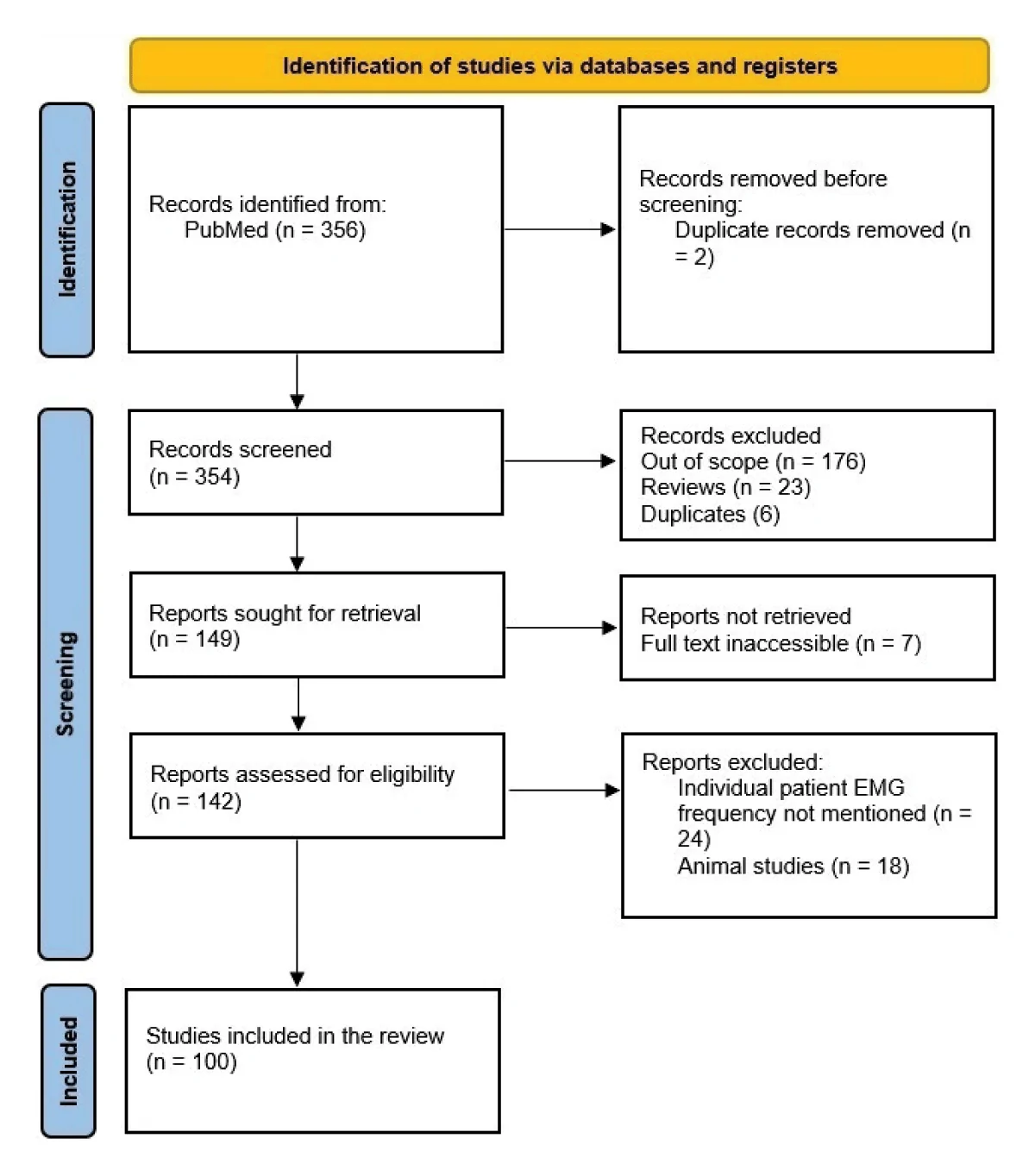
PRISMA flowchart.

### Frequency distribution

The mean tremor frequency across all cases was 13.32 ± 4.15 Hz, with a range of 3–20 Hz (Figure 2). According to the current MDS classification, we grouped 127 patients as having primary OT. 39 had OT plus, and 61 had pseudo-orthostatic tremor. 17 cases could not be definitively classified within the existing framework: 12 showed frequency fluctuations across the 13-Hz threshold, and five exceeded the upper frequency limit of 18 Hz (Table 1). Using our operational clinical grouping, 148 patients were classified as having isolated OT and 96 as having non-isolated presentations (Table 2).

**Figure 2:**
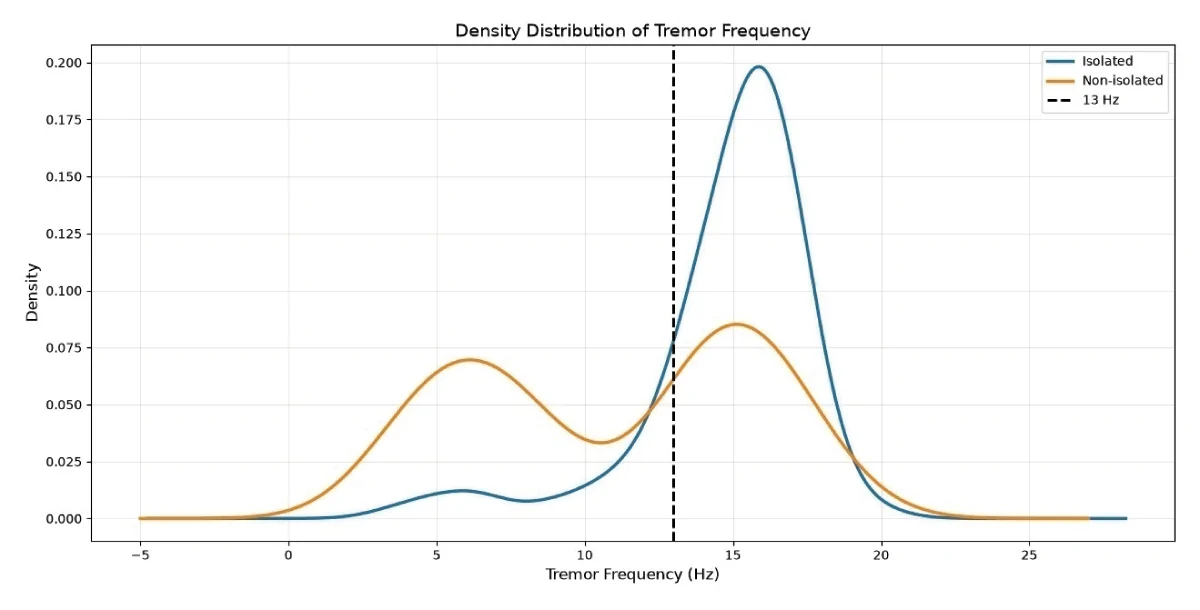
Density plots of tremor frequency for isolated (blue) and non-isolated (orange) OT.

**Table 1:** Clinical spectrum of OT cases according to the MDS classification.


PARAMETER	PRIMARY OT	OT PLUS	PSEUDO OT	UNCLASSIFIABLE (FLUCTUATING FREQUENCY)	UNCLASSIFIABLE (>18 Hz)

N	127	39	61	12	5

Age (SD)	64.13 (11.25)	64.41(13.55)	58.56 (16.03)	61.67 (17.48)	61.80 (13.05)

Sex (M/F)	50/77	14/25	25/36	4/8	3/2

Mean Tremor Frequency in Hz (SD)	15.58 (1.26)	15.46 (1.41)	6.82 (2.36)	13.12 (1.17)	18.92 (0.71)

Familial history of OT	13	2	2	0	0


**Table 2:** Clinical spectrum of OT cases based on isolated/non-isolated phenotype.


PARAMETER	ISOLATED OT	NON-ISOLATED OT

N	148	96

Age (SD)	63.97 (11.35)	60.51 (15.96)

Sex (M/F)	60/88	36/60

Mean Tremor Frequency in Hz (SD)	14.80 (2.82)	11.03 (4.81)

≥13 Hz/<13 Hz	129/19	49/47

Familial history of OT	12	5


Patients with non-isolated OT had significantly lower tremor frequencies compared to those with isolated OT (11.03 Hz vs. 14.80 Hz, p < 0.001). In contrast, age and sex were not associated with tremor frequency or phenotype. A positive correlation was observed between tremor frequency and reported disease duration (Spearman’s ρ = 0.366, p < 0.001).

Receiver operating characteristic analysis showed that tremor frequency had the discriminatory ability to distinguish isolated from non-isolated OT (AUC = 0.72, p < 0.001, 95% CI, 0.60–0.85). The optimal frequency threshold identified using the Youden index was 10 Hz with a sensitivity of 45% and specificity of 93%.

To determine whether tremor frequency independently predicted clinical phenotype, a multivariable logistic regression model was constructed with non-isolated OT as the dependent variable and age, sex, disease duration, and tremor frequency as covariates. Tremor frequency was the only independent predictor with frequency inversely associated with non-isolated orthostatic tremor (OR = 0.78, 95% CI 0.71–0.85, p < 0.001), whereas age, sex, and disease duration were not significant predictors (Nagelkerke R^2^ = 0.27).

### Conditions associated with OT

Parkinsonism and related disorders were most commonly associated with OT (n = 38). This was followed by essential tremor, cerebellar disorders and lesions, and unilateral brainstem lesions (midbrain and pons). Non-neurological disorders such as hyperthyroidism and musculoskeletal injury (knee injury) were also associated with OT. The frequency distribution of OT associated with other conditions is presented in Table 3.

**Table 3:** Disorders associated with OT.


ASSOCIATED DISORDERS	TOTAL NUMBER	<13 Hz N (%)	≥13 Hz N (%)	MEAN TREMOR FREQUENCY	SD	MINIMUM FREQUENCY	MAXIMUM FREQUENCY

Parkinson’s disease/Parkinsonism/PSP	38	22 (57.9)	16 (42.1)	10.07	5.171	3	18

Essential tremor	10	3 (30)	7 (70)	13.50	3.752	6.5	19

Cerebellar disorders/lesions	10	2 (20.0)	8 (80.0)	12.73	3.382	4.5	15

Brainstem lesions	6	2 (33.3)	4 (66.7)	12.37	5.258	4.25	17.5

Hyperthyroid states	5	3 (60.0)	2 (40.0)	10.50	4.213	6	15

Musculoskeletal injury	2	1 (50)	1 (50)	10.75	7.425	5.5	16

Hereditary spastic paraplegia	3	1 (33.3)	2 (66.7)	14.00	4.583	9	18

Stiff person syndrome	2	0 (0.0)	2 (100.0)	16.00	0.000	16	16

Dementia	2	0 (0.0)	2 (100.0)	15.00	2.828	13	17

Miscellaneous	18	13 (72.2)	5 (27.8)	8.98	4.502	3.5	16

Isolated OT	148	19 (12.8)	129 (87.2)	14.80	2.821	3.5	20


## Discussion

OT has long occupied an uncertain position within the broader landscape of tremor disorders, initially regarded as a rare and relatively homogeneous condition [1,3]. This review, which synthesizes published individual cases across the clinical spectrum of OT, suggests that the disorder is more heterogeneous than previously thought.

Although the pathophysiology of OT remains incompletely understood, the disorder is widely thought to involve a pathologically active oscillator within the cerebello-thalamo-cortical network [39,113]. Functional imaging and connectivity studies have demonstrated abnormal coupling within lateral cerebellar and supplementary motor area networks in patients with OT, implicating a distributed network rather than a single anatomical node as the likely substrate of rhythmic tremor generation [113]. The ventralis intermedius nucleus of the thalamus occupies a critical position within this circuit, and the therapeutic efficacy of Ventralis intermedius nucleus deep brain stimulation supports its relevance to OT oscillations [52,68].

Our cohort was broadly representative of the OT population described in the literature: predominantly middle-aged to older adults with a female preponderance [3]. However, a meaningful subset of patients could not be assigned to existing categories without ambiguity. Two patterns of nonconformity emerged. In one group, tremor frequency fluctuated considerably across the 13-Hz boundary used to distinguish primary OT from lower-frequency variants, making single-point classification dependent on when the recording was obtained. In a smaller group, tremor frequencies exceeded the upper 18-Hz limit, for which the current criteria designates no formal category.

The finding that some patients fluctuate across the 13-Hz boundary is consistent with a dynamic oscillator model and has precedent in the tremor literature [114,115]. Thompson et al. provided early electrophysiological documentation of patients with primary OT at 14–16 Hz who exhibited intermittent oscillatory episodes at approximately half that frequency [9]. They noted that the primary 16-Hz tremor appeared as a fine ripple beneath the skin because of partial fusion of muscle contractions, whereas the 8-Hz subharmonic produced a more clinically apparent oscillation and worsened the patient’s subjective unsteadiness. Although the mechanism was uncertain, the overall 16-Hz frequency supported a central rather than peripheral origin. The authors proposed that the tremor arose from abnormal feed-forward postural signals required for standing, implicating central oscillatory structures such as the inferior olive or thalamus. Subsequent reports have reinforced the clinical relevance of an 8-Hz subharmonic arising from a 16-Hz primary oscillator, suggesting that subharmonic states may have functional consequences rather than representing electrophysiological artifacts [115]. Multimode oscillatory behaviour is not unique to OT. In Parkinsonian and essential tremor, simultaneous recordings have shown coherent activity at both the tremor frequency and its harmonic, indicating that harmonic and subharmonic modes can operate within the same network [116,117].

Density plots of tremor frequency (Figure 2) showed a bimodal distribution of frequencies for the non-isolated group. The lower and higher frequency clusters of non-isolated OT observed from the plot did not differ distinctly in the spectrum of OT-associated etiologies. However, this does raise the question of why frequencies cluster in a bimodal distribution and whether the two clusters differ in tremor phenomenology. Further studies are needed to validate and explain the same. On the other hand, the majority of the isolated OT patients were found in the ≥13 Hz range. We found that among these patients with isolated OT, those with tremor frequency below 13 Hz had a lower average age than those with higher frequencies (58.78 vs 64.73 years). This raises the possibility that isolated sub-13-Hz OT, in some patients, may represent an early manifestation of neurodegenerative disorders such as Parkinson’s disease, with patients initially presenting with isolated OT before later developing symptoms of neurodegenerative disease, and hence, a non-isolated phenotype with time. Although our study design prevents us from making definitive conclusions, previous studies support this hypothesis. Ganos et al. reported that seven isolated OT cases subsequently developed additional neurological signs, including one patient who developed Parkinson’s disease [19]. In another case series, four patients with sub-13-Hz OT later developed parkinsonism, and all had a marked response to levodopa [10]. We also observed a positive correlation between tremor frequency and disease duration, which potentially may suggest that tremor frequency evolves with disease. Because disease duration and tremor frequency were assessed cross-sectionally, this association should be interpreted carefully.

Although tremor frequency independently distinguished isolated from non-isolated OT, its discriminatory performance was limited. These findings indicate that while tremor frequency is a significant electrophysiological marker, it may be insufficient as a standalone classifier of clinical phenotype. Furthermore, the data-driven exploratory threshold (≈ 10 Hz) differed from the conventional 13 Hz boundary, and approximately 6.97% of EMG-confirmed patients remained unclassifiable using the existing frequency-based criteria. These findings suggest that while tremor frequency remains an important electrophysiological biomarker, rigid frequency thresholds alone incompletely capture the clinical and electrophysiological spectrum of orthostatic tremor.

The distribution of orthostatic tremor (OT) frequencies across associated neurological and systemic conditions demonstrates substantial overlap between traditionally defined clinical categories. While Parkinsonian disorders accounted for the largest subgroup, a nearly even split between <13 Hz and ≥13 Hz tremors was observed. The frequency distribution was found to be bimodal, similar to the distribution observed for non-isolated OT. Similarly, essential tremor, cerebellar disorders, and brainstem lesions exhibited wide frequency dispersion with substantial proportions of cases exceeding 13 Hz, further supporting a continuous rather than categorical frequency spectrum. Additionally, hyperthyroid states and musculoskeletal injuries, conditions not inherently neurological in nature, also demonstrated both low and high frequency OT [37,51,71,98,106]. Collectively, these findings indicate marked phenotypic overlap across diverse groups of disorders associated with OT.

A small number of published cases fell outside the 18 Hz defined range, raising questions about how strictly existing EMG frequency cutoffs should be applied. Patients with tremor frequencies exceeding 18 Hz otherwise resembled patients with primary OT, supporting the notion that the current upper frequency limit may represent an arbitrary boundary. We also observed that the MDS classification forced every ≥13 Hz patient into one of two categories: Primary OT (Isolated) or OT-plus. This creates a classification gap for non-neurological diseases such as hyperthyroidism and musculoskeletal injuries, which are neither isolated (Primary OT) nor qualify as neurological symptoms (OT plus). However, their causal or contributory relationships remain uncertain [37,106]. The MDS consensus also does not clearly define whether stable neurological sequelae, for example, old polio count as OT-plus [50]. It also does not clarify whether incidental MRI abnormalities should count, or whether unrelated neurological symptoms such as neuropathy secondary to diabetes mellitus qualify as OT-plus, resulting in inconsistent classification [42]. However, we acknowledge that not every neurological comorbidity necessarily has a mechanistic relationship with OT, making it challenging to assign a specific classification category for every possible associated disorder.

Several limitations should be acknowledged. The available evidence is derived largely from case reports and case series, which may disproportionately reflect unusual or atypical presentations rather than commonly encountered cases. Reporting bias/publication bias may therefore influence estimates of frequency distribution and associated clinical features. Cases with subharmonics may also be misrepresented if clinicians regard an 8-Hz component as diagnostically insignificant or if they do not report the full frequency range. A related ambiguity is whether the 16-Hz or 8-Hz component should be regarded as the primary frequency, a question with direct implications for classification. In addition, because frequency ranges were converted to mean values for analysis, within-patient variability may have been reduced.

Our review found that the large majority of published cases could be accommodated within the current MDS classification. However, a small subset demonstrated frequency fluctuations across the 13-Hz boundary or frequencies above 18 Hz, highlighting limitations at the margins of the current frequency-based framework. Although tremor frequency remains an important diagnostic feature of OT, our findings indicate that rigid frequency thresholds alone do not adequately distinguish isolated from non-isolated presentations or account for patients with frequency variability, boundary-spanning frequencies, or frequencies outside the currently accepted range. The substantial overlap in tremor frequencies across diverse neurological and non-neurological conditions further suggests that frequency should be interpreted within its clinical context rather than as the primary determinant of disease subtype.

## Supplementary table 1

**Table:** 


SL NO	AUTHOR	YEAR OF PUBLICATION	AGE	SEX	FREQUENCY (HZ)	ISOLATED/NON-ISOLATED	ASSOCIATED CONDITION	MDS CLASSIFICATION

1	Tseng et al. [20]	2025	34	Male	3	Non-isolated	Butane induced encephalopathy, Parkinsonism	Pseudo OT

2	Swinnen et al. [21]	2022	57	Female	4–5	Isolated	-	Pseudo OT

3	Grobe-Einsler et al. [22]	2020	53	Female	14–16	Isolated	-	Primary OT

4	Verriello et al. [23]	2020	82	Male	15–16	Isolated	-	Primary OT

			77	Male	15–16	Isolated	-	Primary OT

5	Yoshii et al. [24]	2020	62	Female	5–6	Isolated	-	Pseudo OT

6	Yoo et al. [25]	2020	69	Female	4–6	Isolated	-	Pseudo OT

7	Chaithra et al. [26]	2019	72	Female	15–16	Non-isolated	Parkinson’s disease	OT Plus

8	Wadhwa et al. [27]	2019	62	Female	17–18	Isolated	-	Primary OT

9	Iovino et al. [28]	2019	23	Male	10.5	Isolated	-	Pseudo OT

10	Santus et al. [29]	2019	73	Female	17	Non-isolated	Frontotemporal dementia	OT Plus

11	Mousele et al. [30]	2018	66	Male	11–12	Isolated	-	Pseudo OT

12	Yoo et al. [31]	2019	48	Male	4–6	Non-isolated	Parkinson’s disease	Pseudo OT

13	Wuehr et al. [32]	2018	62	Male	14.5	Isolated	-	Primary OT

			55	Female	14	Isolated	-	Primary OT

			75	Male	17.5	Isolated	-	Primary OT

			75	Male	14	Isolated	-	Primary OT

			56	Female	17	Isolated	-	Primary OT

			75	Female	16.5	Isolated	-	Primary OT

			66	Female	14	Isolated	-	Primary OT

			75	Male	13.5	Isolated	-	Primary OT

			76	Male	15	Isolated	-	Primary OT

14	Evidente et al. [33]	2018	73	Male	10–14	Isolated	-	Unclassifiable(fluctuating)

			68	Male	5–12	Non-isolated	Essential Tremor	Pseudo OT

15	Shirai et al. [34]	2018	60	Male	4–5	Non-isolated	Machado-Joseph disease	Pseudo OT

16	Ruiz-Julián et al. [35]	2018	75	Female	14	Isolated	-	Primary OT

17	Lehn et al. [36]	2017	68	Male	16	Isolated	-	Primary OT

18	Larrosa Campo et al. [37]	2019	81	Male	14–16	Non-isolated	Thyrotoxicosis	Primary OT

19	Chiang et al. [38]	2017	74	Female	~16	Isolated	-	Primary OT

			73	Male	~16	Isolated	-	Primary OT

20	Vidailhet et al. [39]	2017	46	Female	17	Isolated	-	Primary OT

21	Schöberl et al. [40]	2017	56	Female	17.2	Isolated	-	Primary OT

			64	Female	16	Isolated	-	Primary OT

			72	Male	14	Isolated	-	Primary OT

			73	Male	16.7	Isolated	-	Primary OT

			68	Female	15.5	Isolated	-	Primary OT

			74	Male	14.2	Isolated	-	Primary OT

			73	Male	13.5	Isolated	-	Primary OT

			73	Female	16.5	Isolated	-	Primary OT

			73	Male	14.5	Isolated	-	Primary OT

			73	Female	14.2	Isolated	-	Primary OT

22	Benito-Léon et al. [41]	2016	74	Female	16–18	Isolated	-	Primary OT

			64	Female	10–12	Isolated	-	Pseudo OT

			57	Male	16–18	Isolated	-	Primary OT

			68	Female	16–18	Non-isolated	Parkinson’s disease	OT Plus

			78	Female	16–18	Non-isolated	Parkinson’s disease	OT Plus

			62	Female	12–16	Isolated	-	Unclassifiable(fluctuating)

			37	Female	16–18	Isolated	-	Primary OT

			76	Female	12–16	Isolated	-	Unclassifiable(fluctuating)

			75	Female	12–16	Non-isolated	Parkinson’s disease	Unclassifiable(fluctuating)

			77	Female	12–16	Non-isolated	Parkinson’s disease	Unclassifiable(fluctuating)

			38	Male	16–18	Isolated	-	Primary OT

			81	Female	12–16	Non-isolated	Parkinson’s disease	Unclassifiable(fluctuating)

			65	Female	16–18	Isolated	-	Primary OT

			57	Female	16–18	Isolated	-	Primary OT

23	Blahak et al. [42]	2016	58	Female	15	Non-isolated	Diabetic neuropathy	Primary OT

			72	Male	14	Isolated	-	Primary OT

			73	Female	17	Isolated	-	Primary OT

			67	Male	17	Isolated	-	Primary OT

24	Coleman et al. [43]	2016	61	Female	14	Isolated	-	Primary OT

			74	Female	13–18	Isolated	-	Primary OT

25	Athauda et al. [44]	2017	46	Female	16	Isolated	-	Primary OT

			73	Male	16	Isolated	-	Primary OT

			66	Male	17	Non-isolated	Parkinsonism	OT Plus

26	Contarino et al. [45]	2015	70	Female	16	Isolated	-	Primary OT

27	Erro et al. [46]	2014	63	Female	15	Non-isolated	Hereditary spastic paraparesis	OT Plus

28	Mazzucchi et al. [47]	2014	70	Female	8	Non-isolated	Graves’ disease	Pseudo OT

29	Adebayo et al. [48]	2014	57	Female	16	Isolated	-	Primary OT

30	Vetrugno et al. [49]	2013	77	Female	16	Non-isolated	Stiff Person Syndrome	OT Plus

			55	Female	16	Non-isolated	Stiff Person Syndrome	OT Plus

31	Bhattacharyya et al. [50]	2013	12	Male	15–16	Non-isolated	Poliomyelitis	Primary OT

			7	Male	14–15	Non-isolated	Essential tremor	OT Plus

32	Coffeng et al. [17]	2013	86	Male	10–11	Isolated	-	Pseudo OT

33	Lin et al. [51]	2013	26	Male	8–9	Non-isolated	Graves’ disease	Pseudo OT

34	Lyons et al. [52]	2012	75	Male	13	Isolated	-	Primary OT

35	Virmani et al. [53]	2012	79	Male	13.5	Isolated	-	Primary OT

			75	Female	13.3	Isolated	-	Primary OT

36	Lee et al. [54]	2012	64	Male	11–13	Non-isolated	Spinal Schwannoma	Unclassifiable(fluctuating)

37	Lee et al. [55]	2011	81	Female	4–9	Non-isolated	Parkinson’s disease	Pseudo OT

38	Kumar et al. [56]	2011	60	Male	16	Isolated	-	Primary OT

39	Hegde et al. [57]	2011	69	Male	3–4	Isolated	-	Pseudo OT

40	Yaltho et al. [58]	2011	78	Male	14	Isolated	-	Primary OT

41	Magariños-Ascone et al. [59]	2010	68	Female	12–18	Isolated	-	Unclassifiable(fluctuating)

42	Williams et al. [60]	2010	70	Female	9	Non-isolated	Cerebellar atrophy	Pseudo OT

43	Vetrugno et al. [61]	2010	58	Female	16	Non-isolated	Left midbrain lesion	OT Plus

44	Raudino et al. [62]	2009	59	Male	13–14	Isolated	-	Primary OT

45	Baker et al. [63]	2009	38	Female	8–15	Non-isolated	Multiple Sclerosis	Unclassifiable(fluctuating)

46	Kang et al. [64]	2009	61	Female	6	Non-isolated	Parkinson’s disease	Pseudo OT

			60	Female	6–7	Non-isolated	Parkinson’s disease	Pseudo OT

			61	Female	4–5	Non-isolated	Parkinson’s disease	Pseudo OT

			45	Female	3–4	Non-isolated	Parkinson’s disease	Pseudo OT

			47	Female	16–17	Non-isolated	Parkinson’s disease	OT Plus

47	Ramtahal et al. [65]	2008	78	Male	13–14	Isolated	-	Primary OT

			65	Male	15–16	Isolated	-	Primary OT

			65	Female	15	Isolated	-	Primary OT

48	Wegner et al. [66]	2008	61	Male	16–18	Isolated	-	Primary OT

			61	Female	16–18	Isolated	-	Primary OT

49	Espay et al. [67]	2008	73	Female	15	Isolated	-	Primary OT

			67	Female	15	Isolated	-	Primary OT

50	Guridi et al. [68]	2008	55	Female	16	Isolated	-	Primary OT

51	Trocello et al. [69]	2008	49	Male	16	Isolated	-	Primary OT

			76	Female	17	Isolated	-	Primary OT

			55	Female	17	Isolated	-	Primary OT

			66	Male	16	Isolated	-	Primary OT

			66	Female	15	Isolated	-	Primary OT

			49	Female	15	Isolated	-	Primary OT

			75	Female	16	Isolated	-	Primary OT

			62	Female	16	Isolated	-	Primary OT

			74	Female	16	Isolated	-	Primary OT

			78	Female	17	Isolated	-	Primary OT

			70	Female	18	Isolated	-	Primary OT

			70	Male	15	Isolated	-	Primary OT

52	Stich et al. [70]	2009	57	Female	17.5	Non-isolated	Demyelinating lesion involving the right brainstem, Biclonal gammopathy of undetermined significance	OT Plus

53	Tan et al. [71]	2008	50	Female	14–16	Non-isolated	Graves’ disease	Primary OT

54	Leu-Semenescu et al. [72]	2007	72	Female	18	Non-isolated	Parkinson’s disease	OT Plus

			77	Female	16	Non-isolated	Parkinson’s disease	OT Plus

			79	Male	13.5	Non-isolated	Parkinson’s disease	OT Plus

			75	Female	17	Non-isolated	Parkinson’s disease	OT Plus

			76	Female	7.5–9	Non-isolated	Parkinson’s disease	Pseudo OT

			85	Female	5	Non-isolated	Parkinson’s disease	Pseudo OT

			57	Female	5–6	Non-isolated	Parkinson’s disease	Pseudo OT

			54	Female	4.3–5	Non-isolated	Parkinson’s disease	Pseudo OT

55	Fischer et al. [73]	2007	69	Male	16–17	Isolated	-	Primary OT

			68	Male	16–17	Isolated	-	Primary OT

			63	Male	16–17	Isolated	-	Primary OT

56	Thomas et al. [10]	2007	47	Female	6–7	Non-isolated	Parkinsonism	Pseudo OT

			52	Male	6–7	Non-isolated	Parkinsonism	Pseudo OT

			52	Male	6–7	Non-isolated	Parkinsonism, Kearns-Sayre syndrome	Pseudo OT

			26	Female	6–7	Non-isolated	Parkinsonism	Pseudo OT

57	de Bie et al. [74]	2007	67	Female	16–17	Non-isolated	Progressive supranuclear palsy	OT Plus

58	Krauss et al. [75]	2006	58	Female	15–16	Isolated	-	Primary OT

			72	Male	14	Isolated	-	Primary OT

59	Contarino et al. [76]	2006	68	Male	13	Isolated	-	Primary OT

			68	Male	13	Isolated	-	Primary OT

60	Gilhuis et al. [77]	2005	55	Female	3–5	Non-isolated	Small cell carcinoma of lung	Pseudo OT

61	Karlberg et al. [78]	2005	46	Female	12	Isolated	-	Pseudo OT

			36	Male	8.5	Isolated	-	Pseudo OT

			63	Female	12.5	Isolated	-	Pseudo OT

			64	Female	15.5	Isolated	-	Primary OT

			73	Female	18	Isolated	-	Primary OT

62	Bacsi et al. [79]	2005	59	Female	15.9	Isolated	-	Primary OT

			73	Male	17.3	Isolated	-	Primary OT

			50	Female	15.2	Isolated	-	Primary OT

			57	Female	16.5	Isolated	-	Primary OT

			64	Female	18.1	Isolated	-	Unclassifiable(>18 Hz)

			68	Female	17.3	Isolated	-	Primary OT

			51	Female	17.3	Isolated	-	Primary OT

			69	Female	16.8	Isolated	-	Primary OT

			75	Female	15.6	Isolated	-	Primary OT

			66	Male	15.7	Isolated	-	Primary OT

63	Rodrigues et al. [12]	2005	68	Female	19	Isolated	-	Unclassifiable(>18 Hz)

			70	Female	14	Isolated	-	Primary OT

			74	Female	16	Isolated	-	Primary OT

			53	Male	15	Isolated	-	Primary OT

			63	Female	14	Isolated	-	Primary OT

			65	Female	13	Isolated	-	Primary OT

64	Trip et al. [80]	2003	43	Female	16	Isolated	-	Primary OT

65	Apartis et al. [81]	2001	75	Female	18	Non-isolated	Parkinson’s disease	OT Plus

66	Finkel et al. [13]	2000	76	Male	18–19	Isolated	-	Unclassifiable(>18 Hz)

67	Cavallazi et al. [14]	2000	42	Female	16	Isolated	-	Primary OT

			41	Male	20	Isolated	-	Unclassifiable(>18 Hz)

			59	Female	17	Isolated	-	Primary OT

			48	Female	15	Isolated	-	Primary OT

68	Benito-Leon et al. [82]	2000	68	Male	15	Non-isolated	B12 deficiency	OT Plus

69	Manto et al. [83]	1999	49	Male	14	Non-isolated	Pancerebellar atrophy	OT Plus

			62	Female	15	Non-isolated	Pancerebellar atrophy	OT Plus

			64	Female	14	Non-isolated	Pancerebellar atrophy	OT Plus

70	Wills et al. [84]	1999	74	Male	14.5	Non-isolated	Parkinson’s disease	OT Plus

71	Sander et al. [85]	1998	79	Female	15–17	Isolated	-	Primary OT

			64	Male	15–17	Isolated	-	Primary OT

72	Setta F et al. [86]	1998	48	Male	14	Non-isolated	Cerebellar atrophy	OT Plus

			63	Female	14	Non-isolated	Cerebellar atrophy	OT Plus

			61	Female	15	Non-isolated	Cerebellar atrophy	OT Plus

73	Tsai et al. [87]	1998	58	Female	16	Isolated	-	Primary OT

			68	Female	16	Isolated	-	Primary OT

74	Benito-Leon et al. [88]	1997	49	Female	7–8	Non-isolated	Right pontine lesion	Pseudo OT

			39	Male	14–15	Non-isolated	Left pontine lesion	OT Plus

75	Hanna et al. [89]	1997	33	Female	17.5	Isolated	-	Primary OT

76	Poersch et al. [90]	1994	60	Female	14	Isolated	-	Primary OT

77	Britton et al. [91]	1992	63	Female	16–18	Isolated	-	Primary OT

			25	Female	8–16	Isolated	-	Unclassifiable(fluctuating)

			68	Male	16–17	Isolated	-	Primary OT

			57	Female	15–16	Isolated	-	Primary OT

			47	Female	16–18	Isolated	-	Primary OT

			64	Male	15–16	Isolated	-	Primary OT

78	Willeit et al. [92]	1991	77	Male	14–16	Isolated	-	Primary OT

			57	Male	14–16	Isolated	-	Primary OT

79	FitzGerald et al. [15]	1991	60	Male	18–20	Non-isolated	Essential tremor	Unclassifiable(>18 Hz)

			46	Male	12–14	Non-isolated	Essential tremor	Unclassifiable(fluctuating)

			70	Male	12	Non-isolated	Essential tremor	Pseudo OT

			56	Female	14	Non-isolated	Essential tremor	OT Plus

80	Walker et al. [93]	1990	79	Male	15	Isolated	-	Primary OT

81	Gabellini et al. [94]	1990	68	Female	14–16	Isolated	-	Primary OT

			56	Female	14–16	Isolated	-	Primary OT

			45	Female	14–16	Isolated	-	Primary OT

			75	Female	6–7	Isolated	-	Pseudo OT

			64	Male	6–7	Isolated	-	Pseudo OT

			66	Male	6–7	Isolated	-	Pseudo OT

			59	Male	6–7	Non-isolated	Aqueduct stenosis hydrocephalus	Pseudo OT

			47	Female	6–7	Non-isolated	Chronic relapsing radiculopathy	Pseudo OT

82	Oda et al. [95]	1989	79	Female	4	Non-isolated	Vascular Parkinsonism	Pseudo OT

83	Uncini et al. [96]	1989	73	Male	8–10	Isolated	-	Pseudo OT

			64	Female	16	Isolated	-	Primary OT

84	Papa et al. [97]	1988	63	Female	16	Non-isolated	Essential tremor	OT Plus

			72	Female	16	Non-isolated	Essential tremor	OT Plus

			68	Female	16	Non-isolated	Essential tremor	OT Plus

85	Thompson et al. [9]	1986	55	Male	8–16	Isolated	-	Unclassifiable(fluctuating)

86	Kim et al. [98]	2023	18	Male	5–6	Non-isolated	Knee contusion	Pseudo OT

87	Park et al. [99]	2020	79	Female	4–4.5	Non-isolated	Right pontine lesion	Pseudo OT

			78	Female	6.5	Non-isolated	Parkinsonism	Pseudo OT

			74	Female	8	Non-isolated	Brain lesion	Pseudo OT

			46	Female	9	Non-isolated	Parkinson’s disease	Pseudo OT

			63	Male	13.5–14	Non-isolated	Restless leg syndrome	OT Plus

			62	Female	6	Non-isolated	Drug induced parkinsonism	Pseudo OT

			59	Male	5–6	Non-isolated	Tardive dyskinesia	Pseudo OT

			68	Female	15	Isolated	-	Primary OT

			49	Male	4–5	Non-isolated	Arteriovenous malformation	Pseudo OT

			65	Female	4	Non-isolated	REM sleep behaviour disorder	Pseudo OT

			67	Male	8–8.5	Non-isolated	Parkinsonism with Normal Pressure hydrocephalus	Pseudo OT

			58	Female	16	Isolated	-	Primary OT

			72	Male	13	Non-isolated	Dementia	OT Plus

			61	Male	13	Isolated	-	Primary OT

			56	Female	13	Isolated	-	Primary OT

			72	Male	8	Non-isolated	Parkinson’s disease with Essential Tremor	Pseudo OT

			68	Male	6–7	Non-isolated	Essential tremor	Pseudo OT

			65	Female	4–4.5	Non-isolated	Parkinson’s disease	Pseudo OT

88	Comolli et al. [100]	2024	65	Female	6	Non-isolated	Graves’ disease	Pseudo OT

89	Li et al. [101]	2022	22	Male	6–8	Non-isolated	Hyperphenylalaninemia	Pseudo OT

90	Lv et al. [102]	2022	67	Male	16	Non-isolated	Primary malignant melanoma of the spinal cord	OT Plus

91	Bhattacharyya et al. [103]	2003	70	Male	18	Isolated	-	Primary OT

92	Jones et al. [104]	2011	65	Male	16	Isolated	-	Primary OT

93	Manrique-Huarte et al. [105]	2012	65	Female	14–16	Isolated	-	Primary OT

			69	Female	2–9	Non-isolated	Psychogenic dizziness	Pseudo OT

			45	Female	10	Isolated	-	Pseudo OT

			63	Female	8	Non-isolated	Migraine with aura	Pseudo OT

94	Yague et al. [106]	2011	47	Female	16	Non-isolated	Surgery to left meniscus following trauma	Primary OT

95	Sanitate et al. [107]	1993	72	Female	14–15	Non-isolated	Head trauma, demyelinating lesions in the periventricular region and subcortical white matter, Ischemic changesin the right pons and the right cerebellar hemisphere	OT Plus

96	Marechal et al. [108]	2024	73	Male	14	Non-isolated	Restless leg syndrome, PSP	OT Plus

97	Myers et al. [109]	2003	72	Female	16	Isolated	-	Primary OT

98	Sarva et al. [110]	2016	81	Male	13.8–15.9	Non-isolated	Cerebellar degeneration	OT Plus

			57	Male	13	Non-isolated	Spinocerebellar ataxia	OT Plus

99	Picillo et al. [111]	2017	74	Female	18	Non-isolated	Hereditary spastic paraplegia (SPG31)	OT Plus

			30	Female	9	Non-isolated	Hereditary spastic paraplegia (SPG15)	Pseudo OT

100	Brotman et al. [112]	2000	65	Male	3–4	Non-isolated	Neurosyphillis	Pseudo OT


## Data Availability

The datasets generated and analyzed during the current study are available from the corresponding author on reasonable request.
